# Report on Some Cottage Hospitals in Norfolk and Suffolk

**Published:** 1914-01-17

**Authors:** Henry Burdett


### REPORTS ON
### Hospitals of the United Kingdom.

By SIR HENRY BURDETT, K.C.B., K.C.V.O.

SERIES III.

### SOME COTTAGE HOSPITALS IN NORFOLK AND SUFFOLK.

### THE VICTORIA JUBILEE HOSPITAL, SWAFFHAM, NORFOLK.

This hospital was opened in 1888 in commemoration of Queen Victoria's Jubilee. It has a special interest in the sense that, although it only contains seven beds, the loyalty of the inhabitants of Swaffham caused them to reconstruct the hospital by the addition of a bathroom, an operation theatre, and other improvements, which make it to-day a hospital, where any operation can be performed and the best work done with modern facilities. We found it in perfect order throughout. The present matron is evidently highly capable, but overworked. It is not proper that she should have all the cooking to do as well as the nursing, with no one to assist her night or day. We took the opportunity of seeing the honorary secretary, Mr. Gould, and explained to him the extravagance of not authorising the appointment of a probationer at once, a fact which he recognised and hoped to have remedied at an early meeting of the committee. The hospital, from its situation, is often very cold, and a little money would be well expended in introducing a few radiators throughout the buildings, with some simple system of heating, which can readily be done. This hospital, owing to special circumstances which we need not go into, has gradually accumulated a debt of £50. It has apparently some £750 invested, the savings of twenty years, and having regard to the size of the hospital the invested reserve funds need never exceed £1,250. We are strongly of opinion that a supreme effort should be made to pay off the debt of £50 at once, or it might be properly cleared off by a sale of stock. Once this is done the income, judging by the average expenditure in the years previous to 1913, should suffice to defray the annual cost of maintenance and to keep the institution free from debt.

The committee and people of Swaffham, with the medical staff, are to be congratulated on the progressive spirit which is demonstrated by the present excellent condition of the hospital and of its equipment. To prevent misunderstanding we may explain that our purpose in recommending the immediate liquidation of the debt of £50 is, that, so long as it is carried forward from year to year, frequent appeals are likely to be made on behalf of the hospital. Such continuous demands are apt to give a wrong impression of the policy of a committee, who may earn the reputation of being always begging, a danger which should be obviated in the case of the Swaffham Hospital.

### THETFORD COTTAGE HOSPITAL.

This hospital was established in 1898 and contains seven beds, but it might provide for more patients at a pinch. The site and £400 for building were given by the late Mr. Bidwell. The total subscriptions appear to have amounted to £1,200. An excellent balcony and further extensions of the old buildings on the north side, with additional grounds, were given ten years later by the late Mr. J. Vavasseur, C.B. The hospital has an endowment fund of some £1,700 contributed by Mrs. Tyrrell, Mr. Musker, and Mr. Vavasseur, and upwards of £100 was raised as a memorial to King Edward VII. and invested in Consols in 1911. The former matron, who for eight years had been connected with the hospital, having recently resigned, Miss M. E. Swan was elected to succeed her. Miss Swan was trained at the Birmingham Infirmary under Miss Gibson, and her testimonials from Miss Gibson and from Mr. Jordan Lloyd, F.R.C.S., show her to be a lady of wide experience and practical knowledge in all departments of hospital work. We consider this hospital to be fortunate in having secured the services of so highly qualified a matron, a point of much importance in view of the probability of considerable developments in the amount and character of the work which may have to be undertaken ultimately by cottage hospitals throughout the country.

Miss Swan was unfortunately absent when we visited this hospital on October 24, 1913, but we found it in fair order, though the linen, stores, and a few other things were not as tidy as they no doubt would have been had the new matron been in charge of the hospital. There is a mortuary which could be made an obeject-lesson to inspire proper reverence for the dead, if someone would give a small sum of money towards its improvement. It should be kept strictly to the purpose for which it is intended. We found it in use as a bicycle shed, though there was abundant storeroom elsewhere for the bicycles. It also contained an old shell, which had in it some very insanitary linen or cotton-wool and had no lid. Altogether the place was in a very neglected and dirty condition. Such things should not be. We look to Miss Swan and to the committee to see that the mortuary is, once and for all, improved, always properly kept, and that it is reserved entirely for the purposes for which it was built.

When the gardens of this hospital have been cultivated and put in order it should be one of the most attractive institutions in the county. A balcony extension and the improvements in the operation theatre witness to a desire on the part of the committee to keep the hospital up to date, and to make it fulfil in the best possible way the objects for which it was founded. We are indebted to Mr. G. O. Read, the honorary secretary, for his courteous reception and for so kindly giving us much useful information. The hospital exhibits the deep interest taken in its welfare by a former member of the staff and his wife, who seem to have collected a library for the use of the patients, and also to have given much time and shown much interest in its welfare.

### MILDENHALL COTTAGE HOSPITAL.

This hospital has had forty-five years of useful existence, and occupies what was formerly, we understand, a public-house. It is not, nor does it ever appear to have been conducted as a self-contained, active hospital, dealing with all classes of cases however severe. It confines its work mainly to casualties and minor operations, the more serious cases being sent to Addenbrooke's Hospital at Cambridge. It contains eight beds in some five separate rooms, all of which are well kept; some of them, indeed, we found very winsome and attractive. The kitchens and offices were in good order, and there is an excellent airy and comfortably furnished day room for the use of all the patients. A flower-garden at the side, which is the care and pride of the matron, and a large vegetable garden at the back, which is let off, can both apparently be used when the patients desire to walk in either. It is a pity that the whole garden is not under the control of the cottage hospital authorities, but the cost of labour is considered too heavy a charge by the committee and so it is let. This hospital has no mortuary, deaths being necessarily very infrequent. For some reason, which, having regard to the efficient condition of the hospital, we must attribute to the indifference of the inhabitants of Mildenhall, the subscriptions show a tendency to fall away, a fact which is not creditable to the people of Mildenhall or to the district which the hospital serves. We would suggest that the committee should sound an alarm and encourage the people to visit the hospital and see what a nice place it is. Thus it should be possible gradually to win their sympathies and interest and so to secure an ample amount of personal service from the more wise and intelligent of the inhabitants, apart altogether from their social position. Personal service supplies the means whereby the whole community may awake and become alive to the privilege of praising God for the blessing of health, by giving a little of their time each year in the cause of the sick, and for the benefit of their most interesting little hospital.

Miss F. M. Turner, who has been here eight years and was formerly at Ancoats Hospital, Manchester, where she was trained, should win the sympathy, especially of the women, throughout the district, who might properly show their appreciation by extending their hospitality and friendship to a solitary worker who at times must, we fear, find the duties of her office somewhat lonesome. She has, we are glad to say, the companionship of some exceedingly well-kept birds and of a fine collie dog, but a little active human sympathy and expressed appreciation for excellent services rendered during many years to the sick of Mildenhall, would, we are confident, be a great help to her in her work and prove precious also in many ways. It is interesting to note that the honorary treasurer, Mr. Oddy F. Read, is a brother of the honorary secretary of Thetford Hospital, showing how stimulating and helpful indulgence in good works through honorary service may become in a family, where it has once taken root.

**The Bury St. Edmunds and the Huntingdon County Hospitals will be reported on next week.**

